# Sociodemographic correlates of colorectal cancer screening completion among women adherent to mammography screening guidelines by place of birth

**DOI:** 10.1186/s12905-022-01694-1

**Published:** 2022-04-21

**Authors:** Deeonna E. Farr, Leslie E. Cofie, Alison T. Brenner, Ronny A. Bell, Daniel S. Reuland

**Affiliations:** 1 Department of Health Education and Promotion, College of Health and Human Performance, East Carolina University, 2307 Carol G. Belk Building, Mail Stop 529, Greenville, NC 27858 USA; 2 Department of Medicine, Department of General Medicine and Clinical Epidemiology, University of North Carolina at Chapel Hill, Chapel Hill, NC 27599 USA; 3 Department of Social Sciences and Health Policy, Division of Public Health Sciences, Wake Forest School of Medicine, Medical Center Boulevard, Winston-Salem, NC 27157 USA; 4 Lineberger Comprehensive Cancer Center, University of North Carolina at Chapel Hill, Chapel Hill, NC 27599 USA

**Keywords:** Female, Early detection of cancer, Colorectal neoplasms, Breast neoplasms, Demography, Mass screening

## Abstract

**Introduction:**

Colorectal cancer screening rates in the U.S. still fall short of national goals, while screening rates for other cancer sites, such as breast, remain high. Understanding characteristics associated with colorectal cancer screening among different groups of women adherent to breast cancer screening guidelines can shed light on the facilitators of colorectal cancer screening among those already engaged in cancer prevention behaviors. The purpose of this study was to explore which demographic characteristics, healthcare access factors, and cancer-related beliefs were associated with colorectal cancer screening completion among U.S. and foreign-born women adherent to mammography screening recommendations.

**Methods:**

Analyses of the 2015 National Health Interview Survey were conducted in 2019. A sample of 1206 women aged 50–74 who had a mammogram in the past 2 years and were of average risk for colorectal cancer was examined. Logistic regression was used to determine demographic, health service, and health belief characteristics associated with colorectal cancer screening completion.

**Results:**

Fifty-five percent of the sample were adherent to colorectal cancer screening recommendations. Women over the age of 65 (AOR = 1.76, 95% CI 1.06–2.91), with any type of health insurance, and who were bilingual (AOR = 3.84, 95% CI 1.83–8.09) were more likely to complete screening, while foreign-born women (AOR = 0.53, 95% CI 0.34–0.83) were less likely. Cancer-related beliefs did not influence adherence. Stratified analyses by nativity revealed additional associations.

**Conclusions:**

Demographic and health service factors interact to influence colorectal cancer screening among women completing breast cancer screening. Colorectal cancer screening interventions targeting specific underserved groups and financing reforms may enhance women’s colorectal cancer screening rates.

**Supplementary Information:**

The online version contains supplementary material available at 10.1186/s12905-022-01694-1.

## Background

Despite national efforts to address colorectal cancer (CRC) mortality in the United States (U.S.) by increasing screening rates, many populations have not achieved national CRC screening goals [1–3]. Data indicate that individuals adhering to screening guidelines for one cancer site are likely to be adherent across multiple cancer sites. However, CRC test completion rates are among the lowest of all cancers with population-level screening guidelines [3–8]. Among women, CRC screening has been associated with breast cancer (BrCa) screening adherence [1, 4–6, 9–12], but women consistently complete mammography at higher rates [8, 13–15].

While women’s BrCa and CRC screening rates differ, similar demographic characteristics (education, income, race) and health service factors (health insurance coverage, lack of a usual source of care, provider recommendations) are associated with non-adherence for both cancer sites [16–19]. Even when controlling for these factors, differences in screening adherence emerge by nativity status [20, 21]. Foreign-born women who are citizens and longer-term residents complete BrCa screening at higher rates and CRC screening at lower rates than their U.S.-born counterparts [20, 21].

In addition to differences between CRC and BrCa screening completion by nativity, CRC has multiple approved testing modalities compared to one for BrCa, and health beliefs such as perceived risk are more consistently associated with CRC screening behavior compared to mammography [22–28]. Together, these factors may explain lower CRC screening rates among women. Yet, few studies use national data to explore which characteristics are associated with differences in women’s screening behavior for both cancers [4, 29, 30]. As recommended screening ages in the U.S. almost completely overlap for both cancer sites, understanding facilitators of CRC screening among a national sample of U.S. and foreign-born women completing mammography can yield important insights to shape CRC screening approaches for both populations [22, 31–33]. The purpose of this study was to examine what demographic characteristics, health system factors, and cancer beliefs were associated with CRC screening completion in a national sample of women completing breast cancer screening. Secondarily, we sought to examine how these associations with CRC screening adherence varied by place of birth.

## Methods

### Data source

The National Health Interview Survey (NHIS) is a nationally representative cross-sectional household survey of the U.S. civilian non-institutionalized population, based on a complex multistage clustered sample design. Through in-person interviews, demographic and health information is collected from household members using family, person, and sample adult modules. Additional details about the survey design and sampling methodology are available at https://www.cdc.gov/nchs/nhis/index.htm. The 2015 NHIS was used for this analysis as it contains a complete cancer control supplemental module which includes information on CRC family history and chronic conditions needed to determine respondents’ CRC risk. The response rate for 2015 was 55.2% [34].

### Participants

The analytic sample consisted of women aged 50–74 years, with the following inclusion criteria: BrCa free, completed a screening mammogram in the past two years, and were of average BrCa risk (no first degree relative with BrCa or ovarian cancer) and CRC risk (no first degree relative with CRC cancer, and no history of polyps or inflammatory bowel disease). The East Carolina University Institutional Review Board exempted this study from review as it was a secondary analysis of publicly available data.

### Measures

Respondents were defined as adherent to CRC screening if they reported completion of fecal occult blood testing in the past 12 months, a sigmoidoscopy in the past 5 years, or a colonoscopy within the past 10 years for screening purposes [22, 34]. All response options on the survey questions were closed-ended and treated as categorical. As Medicare covers cancer screening procedures at no cost to its enrollees who are 65 years and older, two age groups were created for analysis based on Medicare eligibility: 50–64 years old and 65 years or older [35]. Race and ethnicity were assessed in a single question, and all education levels above college graduate were collapsed into one category. For marital status, widowed, divorced, and separated were collapsed into one category, and health insurance types not listed as private, Medicaid, or Medicare were labeled as “Other.” Both BrCa and CRC perceived risk were collected using single-item assessing whether respondents believed they were more, less, or as likely to be diagnosed with each type of cancer as the general population. All variables in this analysis were selected apriori based on previous literature.

### Analysis

Bivariate associations between sociodemographic, health care access, perceived BrCa risk, perceived CRC risk variables, and colorectal cancer screening adherence were examined using chi-square tests. Multivariate logistic regression analyses stratified by nativity were constructed to examine the factors associated with CRC adherence. Statistical tests were 2-sided, and a *p* value < 0.05 was considered statistically significant. Analyses were weighted due to the complex survey sampling methods used in the NHIS and conducted in 2019 using SAS® 9.4 [34, 36].

## Results

The final analytic sample consisted of 1206 respondents aged 50–74 who had completed BrCa screening according to United States Preventive Services Task Force (USPSTF) guidelines (see Table 1). Half of the total sample completed CRC screening (55%), and colonoscopy was the most frequently used test (50%). There was a significant difference in CRC screening by place of birth (*p* < 0.01) in that U.S.-born women reported higher screening adherence than their foreign-born counterparts (58% vs. 46%). U.S.-born women reported higher rates of colonoscopy usage compared to foreign-born women (52% vs. 42%). Sociodemographic characteristics of the sample stratified by CRC screening status are included in Additional file 1.   After adjusting for sociodemographic factors, foreign-born women were less likely to report screening adherence than U.S.-born women (AOR: 0.53, CI 0.34–0.83) (see Table 2). Also, older age (AOR: 1.76, CI 1.06–2.91), equal Spanish and English fluency compared with English only fluency (AOR: 3.84, CI 1.83–8.09), and having any type of health insurance were associated with screening adherence. In stratified regression models, older age (AOR: 2.70, CI 1.37–5.34), having private, Medicare, or other forms of insurance were associated with colorectal cancer screening among U.S.-born women. Among foreign-born women, Black race (AOR: 3.69, CI 1.22–11.21), health insurance coverage, and bilingual (AOR: 3.03, CI 1.08–8.54) or mostly Spanish fluency (AOR: 3.11, CI 1.03–9.42) were associated with screening adherence.

**Table 1:** Sociodemographic characteristics of women adherent to breast cancer guidelines, by place of birth, NHIS 2015

	Total	Place of birth n (%)		
		US-born	Foreign-born	*p* value
*CRC screening adherent*				
Yes	665 (55.52%)	515 (58.26%)	150 (46.46%)	0.0005
No	541 (44.48%)	374 (41.74%)	167 (53.54%)	
*Types of CRC screening*				
Sigmoidoscopy				
Yes	16 (1.47%)	9 (1.27%)	7 (2.13%)	0.2910
No	1182 (98.53%)	875 (98.73%)	307 (97.87%)	
Colonoscopy				
Yes	602 (50.33%)	470 (52.77%)	132 (42.21%)	0.0015
No	595 (49.67%)	414 (47.23%)	181 (57.49%)	
Fecal occult blood test				
Yes	124 (10.81%)	94 (11.31%)	30 (9.16%)	0.2901
No	1078 (89.19%)	793 (88.69%)	285 (90.84%)	
*Personal characteristics*				
Age				
50–64	841 (70.98%)	630 (72.37%)	211 (66.39%)	0.056
65+	365 (29.02%)	259 (27.63%)	106 (33.61%)	
Race/ethnicity				
White	607 (58.38%)	571 (70.80%)	36 (17.22%)	< 0.001
Hispanic	245 (16.14%)	65 (5.56%)	180 (51.13%)	
Black	248 (18.03%)	220 (20.76%)	28 (8.99%)	
Asian	106 (7.48%)	33 (2.88%)	73 (22.67%)	
Degree				
No high school degree	193 (12.82%)	67 (6.24%)	126 (34.59%)	< 0.001
High school degree	289 (22.90%)	218 (23.42%)	71 (21.19%)	
Some college/associate degree	364 (30.80%)	311 (34.81%)	53 (17.53%)	
College degree or higher	358 (33.48%)	292 (35.53%)	66 (26.69%)	
Federal poverty level				
≤ 138%	280 (19.48%)	168 (16.22%)	112 (30.27%)	< 0.001
139–200%	126 (9.38%)	83 (8.28%)	43 (13.02%)	
210–400%	328 (26.95%)	243 (27.55%)	85 (24.96%)	
≥ 410%	472 (44.19%)	395 (47.95%)	77 (31.75%)	
Marital status				
Married	593 (53.56%)	428 (53.30%)	165 (54.42%)	0.411
Widowed/divorced/separated	469 (36.81%)	345 (36.51%)	124 (37.81%)	
Single	140 (9.63%)	112 (10.19%)	28 (7.78%)	
Region				
Northeast	218 (18.72%)	134 (15.75%)	84 (28.57%)	< 0.001
North Central/Midwest	204 (18.41%)	172 (20.39%)	32 (11.86%)	
South	439 (40.00%)	353 (43.38%)	86 (28.83%)	
West	345 (22.87%)	230 (20.48%)	115 (30.74%)	
Language spoken				
English	796 (69.65%)	749 (85.41%)	47 (17.54%)	< 0.001
Mostly English	154 (13.44%)	105 (11.75%)	49 (10.02%)	
Only Spanish/other language	137 (9.34%)	6 (0.65%)	131 (38.09%)	
Mostly Spanish	48 (3.05%)	0 (0.00%)	48 (13.14%)	
Spanish and English equally	71 (4.51%)	29 (2.19%)	42 (12.19%)	
*Place of birth*				
Foreign-born	317 (23.22%)	–	–	–
U.S.-born	889 (76.78%)	–	–	–
Insurance				
Private	614 (55.96%)	468 (57.55%)	146 (50.73%)	< 0.001
Medicaid	111 (7.33%)	70 (6.65%)	41 (9.55%)	
Medicare	158 (12.24%)	109 (11.10%)	49 (16.01%)	
Dual eligible	62 (4.01%)	33 (2.84%)	29 (8.24%)	
Other	199 (16.61%)	172 (18.53%)	27 (8.90%)	
None	59 (3.76%)	34 (2.91%)	25 (6.57%)	
Breast cancer risk				
More likely to get cancer	48 (4.72%)	33 (4.35%)	15 (5.67%)	0.460
Less likely	589 (49.80%)	445 (50.47%)	144 (47.54%)	
About as likely	501 (45.49%)	364 (45.19%)	137 (46.50%)	
CRC risk				
More likely to get cancer	17 (1.03%)	7 (0.57%)	10 (2.56%)	0.008
Less likely	632 (56.55%)	479 (57.62%)	153 (52.95%)	
About as likely	475 (42.42%)	343 (41.80%)	132 (44.49%)

**Table 2:** Factors associated with CRC screening adherence among women adherent to breast cancer screening, NHIS 2015

	CRC screening adherence			
	Unadjusted OR (95% CI)	Adjusted OR (95% CI) Overall	Adjusted OR (95% CI) US-born	Adjusted OR (95% CI) Foreign-born
*Age*				
50–64	Ref	Ref	Ref	Ref
65+	**1.82 (1.34–2.47)**	**1.76 (1.06–2.91)**	**2.70 (1.37–5.34)**	0.57 (0.28–1.16)
*Race*				
White	Ref	Ref	Ref	Ref
Hispanic	**0.73 (0.55–0.98)**	1.01 (0.59–1.72)	1.63 (0.79–3.38)	0.82 (0.27–2.50)
Black	0.94 (0.70–1.26)	0.95 (0.50–1.80)	1.10 (0.76–1.59)	**3.69 (1.22–11.21)**
Asian	0.68 (0.43–1.06)	1.28 (0.73–2.23)	0.65 (0.30–1.41)	1.05 (0.43–2.56)
*Degree*				
No high school degree	Ref	Ref	Ref	Ref
High school degree	1.21 (0.84–1.74)	1.07 (0.67–1.74)	1.13 (0.63–2.01)	1.23 (0.54–2.81)
Some college/associated degree	**1.44 (1.01–2.06)**	1.39 (0.87–2.23)	1.64 (0.92–2.91)	1.22 (0.45–3.34)
College degree	**1.67 (1.16–2.34)**	1.37 (0.79–2.35)	1.47 0.76–2.83)	1.52 (0.58–4.03)
*Federal poverty level*				
≤ 138%	Ref	Ref	Ref	Ref
139–200%	0.91 (0.59–1.41)	0.80 (0.48–1.33)	0.77 (0.39–1.51)	0.92 (0.40–2.09)
210–400%	1.00 (0.7–1.44)	0.93 (0.60–1.46)	0.92 (0.52–1.64)	0.95 (0.47–1.93)
≥ 410%	**1.50 (1.09–2.08)**	1.43 (0.88–2.35)	1.55 (0.82–2.93)	1.13 (0.54–2.34)
*Insurance*				
Private	**5.19 (2.44–11.03)**	**3.84 (1.83–8.09)**	**3.32 (1.33–8.27)**	**6.94 (1.42–33.88)**
Medicaid	**3.45 (1.53–7.77)**	**2.99 (1.33–6.76)**	2.31 (0.82–6.49)	**6.91 (1.36–35.13)**
Medicare	**7.39 (3.29–16.61)**	**4.15 (1.73–9.94)**	**2.95 (1.02–8.54)**	**18.65 (2.85–122.12)**
Dual eligible	**5.71 (2.15–15.20)**	**3.48 (1.17–10.33)**	2.03 (0.48–8.54)	**20.47 (3.36–124.96)**
Other	**9.16 (4.16–20.15)**	**5.11 (2.10–12.42)**	**2.95 (1.02–8.54)**	**41.51 (5.64–305.55)**
None	Ref	Ref	Ref	Ref
*Marital status*				
Married	Ref	Ref	Ref	Ref
Widowed/divorced/separated	1.08 (0.84–1.41)	1.08 (0.79–1.48)	1.20 (0.82–1.76)	0.97 (0.54–1.72)
Single	1.15 (0.75–1.77)	1.36 (0.83–2.23)	1.74 (0.97–3.10)	0.77 (0.31–1.96)
*Region*				
Northeast	Ref	Ref	Ref	Ref
North Central/Midwest	0.87 (0.55–1.35)	1.01 (0.60–1.70)	1.24 (0.70–2.20)	0.83 (0.33–2.09)
South	0.86 (0.58–1.27)	0.80 (0.52–1.22)	0.82 (0.51–1.320	0.68 (0.27–1.74)
West	0.86 (0.58–1.28)	0.89 (0.56–1.41)	0.68 (0.41–1.12)	1.38 (0.58–3.28)
*Language spoken*				
English	Ref	Ref	Ref	Ref
Mostly English	1.07 (0.77–1.50)	1.04 (0.62–1.74)	1.07 (0.73–1.59)	0.91 (0.33–2.46)
Only Spanish/other language	**0.55 (0.38–0.80)**	1.81 (0.53–1.23)	0.26 (0.04–1.57)	1.37 (0.57–3.29)
Mostly Spanish	0.84 (0.47–1.51)	0.91 (0.57–1.44)	–	**3.11 (1.03–9.42)**
Spanish and English equally	1.16 (0.73–1.83)	**3.84 (1.83–8.09)**	–	**3.03 (1.08–8.54)**
*Place of birth*				
Foreign-born	**0.62 (0.48–0.81)**	**0.53 (0.34–0.83)**	–	–
U.S.-born	Ref	Ref	–	–
*Breast cancer risk*				
More likely to get cancer	Ref	Ref	Ref	Ref
Less likely	0.72 (0.40–1.32)	0.56 (0.27–1.16)	0.50 (0.21- 1.18)	0.72 (0.16–3.26)
About as likely	0.61 (0.33–1.11)	0.56 (0.27–1.16)	0.52 (0.22–1.23)	0.48 (0.13–1.78)
*CRC risk*				
More likely to get cancer	1.68 (0.61–4.62)	Ref	Ref	Ref
Less likely	1.41 (0.51–3.89)	2.01 (0.70–5.73)	2.70 (0.59–12.31)	1.42 (0.35–5.84)
About as likely	Ref	1.82 (0.62–5.32)	2.05 (0.43–9.80)	2.16 (0.60–7.86)

Bolded AORs (95% CI) represent significant findings. All analyses were weighted to account for sampling

## Discussion

Through this analysis, we examined which factors were associated with CRC screening completion in a sample of women already adherent to screening recommendations for another cancer site (breast). Fifty-five percent of women completing mammography reported a recent CRC screening test. Neither perceived BrCa nor CRC risk were found to influence CRC test completion. Multiple demographic and health service factors were associated with CRC screening adherence, but these factors varied by nativity.

This analysis of NHIS data revealed differences in CRC and BrCa screening behavior similar to research using regional samples or claims data. Other studies demonstrate similar differences, with one examination of women with private insurance and/or Medicaid finding that 70% of women completing at least one mammogram between 2010 and 2015 also completed a CRC screening test [4, 6, 30]. However, this data includes CRC survivors and others at elevated CRC risk whose behavior should not be evaluated using screening guidelines for average-risk populations [22, 30, 37]. As our analysis was restricted to women of average risk for both cancers, our findings present a more realistic estimate of the difference in adherence rates.

Despite previous research suggesting perceived cancer risk as a correlate of screening adherence, neither BrCa nor CRC risk perceptions were associated with test completion in this sample [23, 25–28]. While perceived risk is the behavioral construct with the most consistent relationship to CRC test completion, evidence from a study by Hay et al. indicates that CRC screening is positively correlated with perceived risk in analyses of prospective data, not cross-sectional data [38]. The cross-sectional nature of the NHIS may explain the lack of association in this sample [38]. Additionally, it’s possible that other health beliefs and attitudes not measured in this survey may influence CRC screening in this group. International studies of this topic report lack of perceived benefits of and negative attitudes towards CRC screening procedures as possible barriers to CRC screening among those completing mammography [39, 40]. U.S.-based studies assessing CRC screening behaviors and beliefs of U.S.-born women waiting for mammography procedures found that an endorsement of the perceived benefits of screening and high levels of self-efficacy to be positively associated with CRC adherence [29, 41]. Among those same women high levels of perceived barriers were inversely associated with screening adherence [29, 41]. Given that many of the international studies took place in countries which provide universal healthcare coverage and standardized reminders to complete screening, these attitudes may not function in the same way for US populations where awareness of screening guidelines and financial concerns play an important role.

Multiple sociodemographic characteristics were associated with CRC screening adherence, but the impact of these characteristics varied by nativity. Among U.S.-born women, those aged 65 and older, with Medicare, private or other types of health insurance were more likely to complete CRC screening. Our findings are in conflict with studies of CRC screening only in women, as income, race, education, in addition to age and type of healthcare coverage are known influences on screening adherence [20]. Meanwhile for foreign born women, Black race, having any health insurance and speaking mostly Spanish or being fluent in both Spanish and English was associated with CRC screening completion. Interactions between proxy measures of acculturation, including length of time in the U.S., education level, and language spoken were examined to explain intergroup differences, but the findings were not statistically significant.

Examinations of CRC screening alone report that foreign-born women have lower adherence rates than their U.S.-born counterparts regardless of length of time in the U.S. [20].

Studies examining both BrCa and CRC screening among Black immigrant women are limited, and investigations of CRC screening adherence levels in this population report mixed results [17, 20, 42–46]. Similarly, few large studies investigate both BrCa and CRC screening in Latina populations [20, 43, 47]. Our findings are in opposition to most of the CRC screening-only literature, which indicates lower CRC screening rates among Black Americans, primarily Spanish speaking, and bilingual Latina immigrants [47, 48]. In respect to Latinas, our findings are consistent with Costas-Muñiz et al. who found that foreign-born, Spanish-speaking, and bilingual Latinos were more likely to complete colonoscopy compared to U.S.-born or English-speaking Latinos in New York City [49]. The authors concluded that the increased availability of CRC screening in the U.S. compared to participants’ home countries may encourage screening behaviors, while medical mistrust and other healthcare experiences may reduce US-born and acculturated foreign-born Latinos’ desire to complete screening [49].

Patterns of CRC screening adherence also vary by type of health insurance and with important differences within nativity status. Foreign-born women with any type of health insurance were more likely to complete CRC screening; however, this was not the case among U.S.-born women with Medicaid or who were dual Medicaid–Medicare eligible. Additionally, U.S.-born women over the age of 65 were more likely to complete CRC screening compared to their younger counterparts, but no age-related differences in CRC adherence were found among foreign-born women. This is likely due to U.S.-born women having access to Medicare after age 65, whereas depending on citizenship and other factors, many foreign-born women are not eligible for Medicare. CRC screening adherence is known to vary by health insurance status, but few studies examine how nativity shapes the types of insurance available and the subsequent impact on screening completion.

In the U.S., financing continues to be a key driver of screening adherence across cancer sites [30, 50, 51]. While screening mammography is a free preventive service, the out-of-pocket costs associated with specific CRC screening modalities, in particular colonoscopy, vary by insurance type [30, 50, 51]. Among women adherent to CRC screening guidelines in our sample, US-born women completed colonoscopy at higher rates than foreign-born women (52.77% vs. 42.21%). Higher colonoscopy rates in this population represent an increased cost burden. Higher CRC screening costs in combination with greater awareness and availability of free and low-cost BrCa screening programs illustrate how variation in policies that dictate promotion and financing of prevention behaviors for specific cancer sites contribute to the difference in women’s BrCa and CRC screening rates [15, 52–55].

Our findings were limited in that the NHIS consists of self-reports of screening adherence, a limited set of cancer-related beliefs and attitudes, and the absence of information on providers’ CRC screening recommendations. Lastly, the analysis was restricted to 1 year of data collection as the variables needed for CRC risk assessment were not available in the NHIS Cancer Control modules for 2018, 2013, or 2010. However, our analysis has several strengths such as the use of a national data set, the inclusion of standardized psychosocial, demographic, and healthcare access variables, and a focus on individuals of average BrCa and CRC risk).

## Conclusion

Examining CRC screening among women completing mammography demonstrates complex relationships between demographic factors, healthcare access, and CRC test choice. Factors such as age, language spoken, and type of health insurance coverage were related to CRC screening adherence. Future studies should seek to clarify how these factors impact CRC screening behaviors in subpopulations, such as non-immigrant and immigrant women, to inform the development of policies to facilitate CRC screening in these groups. As CRC screening is now recommended for younger individuals (45–49 years of age), examining drivers of existing cancer screening behaviors may generate insights for pairing cancer control efforts (such as promoting stool-based CRC screening tests at mammography visits). Additionally, research examining screening behaviors across multiple cancer sites may be enhanced by including a more comprehensive set of cancer beliefs in addition to structural factors.

## Supplementary Information

**Additional file 1. Supplemental Table 1.** Sociodemographic characteristics of women adherent to breast cancer guidelines, by colorectal cancer screening status, NHIS 2015.

## Data Availability

All datasets supporting the conclusions of this manuscript are available through the National Health Interview Survey: https://www.cdc.gov/nchs/nhis/index.htm.

## Abbreviations

CRC: Colorectal cancer
U.S.: United States
BrCa: Breast cancer
NHIS: National health interview survey
USPSTF: United States preventive services task force
