# Correction to: Sociodemographic correlates of colorectal cancer screening completion among women adherent to mammography screening guidelines by place of birth

**DOI:** 10.1186/s12905-022-01878-9

**Published:** 2022-08-01

**Authors:** Deeonna E. Farr, Leslie E. Cofie, Alison T. Brenner, Ronny A. Bell, Daniel S. Reuland

**Affiliations:** 1 Department of Health Education and Promotion, College of Health and Human Performance, East Carolina University, 2307 Carol G. Belk Building, Mail Stop 529, Greenville, NC 27858 USA; 2 Department of Medicine, Department of General Medicine and Clinical Epidemiology, University of North Carolina at Chapel Hill, Chapel Hill, NC 27599 USA; 3 Department of Social Sciences and Health Policy, Division of Public Health Sciences, Wake Forest School of Medicine, Medical Center Boulevard, Winston-Salem, NC 27157 USA; 4 Lineberger Comprehensive Cancer Center, University of North Carolina at Chapel Hill, Chapel Hill, NC 27599 USA

## Correction to: BMC Women’s Health (2022) 22:125 10.1186/s12905-022-01694-1

Following publication of the original article [1], there is formatting error in Tables 1 and 2. The Tables 1 and Table 2 should be as shown below:

**Table 1:** Sociodemographic characteristics of women adherent to breast cancer guidelines, by place of birth, NHIS 2015

	Total	Place of birth n (%)		
		US-born	Foreign-born	*p* value
**CRC screening adherent**				
Yes	665 (55.52%)	515 (58.26%)	150 (46.46%)	0.0005
No	541 (44.48%)	374 (41.74%)	167 (53.54%)	
**Types of CRC screening**				
**Sigmoidoscopy**				
Yes	16 (1.47%)	9 (1.27%)	7 (2.13%)	0.2910
No	1182 (98.53%)	875 (98.73%)	307 (97.87%)	
**Colonoscopy**				
Yes	602 (50.33%)	470 (52.77%)	132 (42.21%)	0.0015
No	595 (49.67%)	414 (47.23%)	181 (57.49%)	
**Fecal occult blood test**				
Yes	124 (10.81%)	94 (11.31%)	30 (9.16%)	0.2901
No	1078 (89.19%)	793 (88.69%)	285 (90.84%)	
**Personal characteristics**				
**Age**				
50–64	841 (70.98%)	630 (72.37%)	211 (66.39%)	0.056
65+	365 (29.02%)	259 (27.63%)	106 (33.61%)	
**Race/ethnicity**				
White	607 (58.38%)	571 (70.80%)	36 (17.22%)	< 0.001
Hispanic	245 (16.14%)	65 (5.56%)	180 (51.13%)	
Black	248 (18.03%)	220 (20.76%)	28 (8.99%)	
Asian	106 (7.48%)	33 (2.88%)	73 (22.67%)	
**Degree**				
No high school degree	193 (12.82%)	67 (6.24%)	126 (34.59%)	< 0.001
High school degree	289 (22.90%)	218 (23.42%)	71 (21.19%)	
Some college/associate degree	364 (30.80%)	311 (34.81%)	53 (17.53%)	
College degree or higher	358 (33.48%)	292 (35.53%)	66 (26.69%)	
**Federal poverty level**				
≤ 138%	280 (19.48%)	168 (16.22%)	112 (30.27%)	< 0.001
139–200%	126 (9.38%)	83 (8.28%)	43 (13.02%)	
210–400%	328 (26.95%)	243 (27.55%)	85 (24.96%)	
≥ 410%	472 (44.19%)	395 (47.95%)	77 (31.75%)	
**Marital status**				
Married	593 (53.56%)	428 (53.30%)	165 (54.42%)	0.411
Widowed/divorced/separated	469 (36.81%)	345 (36.51%)	124 (37.81%)	
Single	140 (9.63%)	112 (10.19%)	28 (7.78%)	
**Region**				
Northeast	218 (18.72%)	134 (15.75%)	84 (28.57%)	< 0.001
North Central/Midwest	204 (18.41%)	172 (20.39%)	32 (11.86%)	
South	439 (40.00%)	353 (43.38%)	86 (28.83%)	
West	345 (22.87%)	230 (20.48%)	115 (30.74%)	
**Language spoken**				
English	796 (69.65%)	749 (85.41%)	47 (17.54%)	< 0.001
Mostly English	154 (13.44%)	105 (11.75%)	49 (10.02%)	
Only Spanish/other language	137 (9.34%)	6 (0.65%)	131 (38.09%)	
Mostly Spanish	48 (3.05%)	0 (0.00%)	48 (13.14%)	
Spanish and English equally	71 (4.51%)	29 (2.19%)	42 (12.19%)	
**Place of birth**				
Foreign-born	317 (23.22%)	–	–	–
U.S.-born	889 (76.78%)	–	–	–
**Insurance**				
Private	614 (55.96%)	468 (57.55%)	146 (50.73%)	< 0.001
Medicaid	111 (7.33%)	70 (6.65%)	41 (9.55%)	
Medicare	158 (12.24%)	109 (11.10%)	49 (16.01%)	
Dual eligible	62 (4.01%)	33 (2.84%)	29 (8.24%)	
Other	199 (16.61%)	172 (18.53%)	27 (8.90%)	
None	59 (3.76%)	34 (2.91%)	25 (6.57%)	
**Breast cancer risk**				
More likely to get cancer	48 (4.72%)	33 (4.35%)	15 (5.67%)	0.460
Less likely	589 (49.80%)	445 (50.47%)	144 (47.54%)	
About as likely	501 (45.49%)	364 (45.19%)	137 (46.50%)	
**CRC risk**				
More likely to get cancer	17 (1.03%)	7 (0.57%)	10 (2.56%)	0.008
Less likely	632 (56.55%)	479 (57.62%)	153 (52.95%)	
About as likely	475 (42.42%)	343 (41.80%)	132 (44.49%)

**Table 2:** Factors associated with CRC screening adherence among women adherent to breast cancer screening, NHIS 2015

	CRC screening adherence			
	Unadjusted OR (95% CI)	Adjusted OR (95% CI) Overall	Adjusted OR (95% CI) US-born	Adjusted OR (95% CI) Foreign-born
**Age**				
50–64	Ref	Ref	Ref	Ref
65+	**1.82 (1.34–2.47)**	**1.76 (1.06–2.91)**	**2.70 (1.37–5.34)**	0.57 (0.28–1.16)
**Race**				
White	Ref	Ref	Ref	Ref
Hispanic	**0.73 (0.55–0.98)**	1.01 (0.59–1.72)	1.63 (0.79–3.38)	0.82 (0.27–2.50)
Black	0.94 (0.70–1.26)	0.95 (0.50–1.80)	1.10 (0.76–1.59)	**3.69 (1.22–11.21)**
Asian	0.68 (0.43–1.06)	1.28 (0.73–2.23)	0.65 (0.30–1.41)	1.05 (0.43–2.56)
**Degree**				
No high school degree	Ref	Ref	Ref	Ref
High school degree	1.21 (0.84–1.74)	1.07 (0.67–1.74)	1.13 (0.63–2.01)	1.23 (0.54–2.81)
Some college/associated degree	**1.44 (1.01–2.06)**	1.39 (0.87–2.23)	1.64 (0.92–2.91)	1.22 (0.45–3.34)
College degree	**1.67 (1.16–2.34)**	1.37 (0.79–2.35)	1.47 0.76–2.83)	1.52 (0.58–4.03)
**Federal poverty level**				
≤ 138%	Ref	Ref	Ref	Ref
139–200%	0.91 (0.59–1.41)	0.80 (0.48–1.33)	0.77 (0.39–1.51)	0.92 (0.40–2.09)
210–400%	1.00 (0.7–1.44)	0.93 (0.60–1.46)	0.92 (0.52–1.64)	0.95 (0.47–1.93)
≥ 410%	**1.50 (1.09–2.08)**	1.43 (0.88–2.35)	1.55 (0.82–2.93)	1.13 (0.54–2.34)
**Insurance**				
Private	**5.19 (2.44–11.03)**	**3.84 (1.83–8.09)**	**3.32 (1.33–8.27)**	**6.94 (1.42–33.88)**
Medicaid	**3.45 (1.53–7.77)**	**2.99 (1.33–6.76)**	2.31 (0.82–6.49)	**6.91 (1.36–35.13)**
Medicare	**7.39 (3.29–16.61)**	**4.15 (1.73–9.94)**	**2.95 (1.02–8.54)**	**18.65 (2.85–122.12)**
Dual eligible	**5.71 (2.15–15.20)**	**3.48 (1.17–10.33)**	2.03 (0.48–8.54)	**20.47 (3.36–124.96)**
Other	**9.16 (4.16–20.15)**	**5.11 (2.10–12.42)**	**2.95 (1.02–8.54)**	**41.51 (5.64–305.55)**
None	Ref	Ref	Ref	Ref
**Marital status**				
Married	Ref	Ref	Ref	Ref
Widowed/divorced/separated	1.08 (0.84–1.41)	1.08 (0.79–1.48)	1.20 (0.82–1.76)	0.97 (0.54–1.72)
Single	1.15 (0.75–1.77)	1.36 (0.83–2.23)	1.74 (0.97–3.10)	0.77 (0.31–1.96)
**Region**				
Northeast	Ref	Ref	Ref	Ref
North Central/Midwest	0.87 (0.55–1.35)	1.01 (0.60–1.70)	1.24 (0.70–2.20)	0.83 (0.33–2.09)
South	0.86 (0.58–1.27)	0.80 (0.52–1.22)	0.82 (0.51–1.320	0.68 (0.27–1.74)
West	0.86 (0.58–1.28)	0.89 (0.56–1.41)	0.68 (0.41–1.12)	1.38 (0.58–3.28)
**Language spoken**				
English	Ref	Ref	Ref	Ref
Mostly English	1.07 (0.77–1.50)	1.04 (0.62–1.74)	1.07 (0.73–1.59)	0.91 (0.33–2.46)
Only Spanish/other language	**0.55 (0.38–0.80)**	1.81 (0.53–1.23)	0.26 (0.04–1.57)	1.37 (0.57–3.29)
Mostly Spanish	0.84 (0.47–1.51)	0.91 (0.57–1.44)	–	**3.11 (1.03–9.42)**
Spanish and English equally	1.16 (0.73–1.83)	**3.84 (1.83–8.09)**	–	**3.03 (1.08–8.54)**
**Place of birth**				
Foreign-born	**0.62 (0.48–0.81)**	**0.53 (0.34–0.83)**	–	–
U.S.-born	Ref	Ref	–	–
**Breast cancer risk**				
More likely to get cancer	Ref	Ref	Ref	Ref
Less likely	0.72 (0.40–1.32)	0.56 (0.27–1.16)	0.50 (0.21- 1.18)	0.72 (0.16–3.26)
About as likely	0.61 (0.33–1.11)	0.56 (0.27–1.16)	0.52 (0.22–1.23)	0.48 (0.13–1.78)
**CRC risk**				
More likely to get cancer	1.68 (0.61–4.62)	Ref	Ref	Ref
Less likely	1.41 (0.51–3.89)	2.01 (0.70–5.73)	2.70 (0.59–12.31)	1.42 (0.35–5.84)
About as likely	Ref	1.82 (0.62–5.32)	2.05 (0.43–9.80)	2.16 (0.60–7.86)

Bolded AORs (95% CI) represent significant findings. All analyses were weighted to account for sampling

The original article has been corrected.

