# Supplementary material for: Sociodemographic correlates of colorectal cancer screening completion among women adherent to mammography screening guidelines by place of birth
Source: BMC Womens Health. 2022 Apr 21;22:125. doi: 10.1186/s12905-022-01694-1 (PMC9022316; doi:10.1186/s12905-022-01694-1)
Supplement: Supplementary file 1 — Additional file 1. Supplemental Table 1. Sociodemographic characteristics of women adherent to breast cancer guidelines, by colorectal cancer screening status, NHIS 2015. [file 12905_2022_1694_MOESM1_ESM.docx]

Supplemental Table.  Sociodemographic characteristics of women adherent to breast cancer guidelines, by colorectal cancer screening status, NHIS 2015

|  |  | CRC Screening Status n (%) | | |
| --- | --- | --- | --- | --- |
|  | Total | Adherent | Nonadherent | P-value |
| Age  50-64  65+ | 841 (70.98%)  365 (29.02%) | 420 (65.64%)  245 (34.36%) | 421 (77.65%)  120 (22.35%) | <.001 |
| Race/ Ethnicity  White  Hispanic  Black  Asian | 607 (58.38%)  245 (16.14%)  248 (18.03%)  106 (7.48%) | 350 (60.70%)  122 (14.55%)  143 (18.26%)  50 (6.49%) | 257 (55.43%)  123 (18.13%)  105 (17.74%)  56 (8.70%) | 0.091 |
| Degree  No high school degree  High school degree  Some college/associate degree  College degree or higher | 193 (12.82%)  289 (22.90%)  364 (30.80%)  358 (33.48%) | 92 (10.94%)  153 (21.49%)  211 (31.36%)  208 (36.21%) | 101 (15.16%)  136 (24.67%)  153 (30.10%)  150 (30.06%) | 0.037 |
| Federal Poverty Level  ≤138%  139% - 200%  210% - 400%  ≥410% | 280 (19.48%)  126 (9.38%)  328 (26.95%)  472 (44.19%) | 134 (18.01%)  66 (8.28%)  175 (24.94%)  290 (48.77%) | 146 (21.32%)  60 (10.75%)  153 (29.45%)  182 (38.47%) | 0.005 |
| Marital Status  Married  Widowed/divorced/Separated  Single | 593 (53.56%)  469 (36.81%)  140 (9.63%) | 321 (52.51%)  262 (37.45%)  79 (10.04%) | 272 (54.87%)  207 (36.02%)  61 (9.11%) | 0.713 |
| Region  Northeast  North Central/Midwest  South  West | 218 (18.72%)  204 (18.41%)  439 (40.00%)  345 (22.87%) | 126 (18.55%)  119 (19.42%)  241 (39.45%)  179 (22.58%) | 92 (18.94%)  85 (17.16%)  198 (40.68%)  166 (23.22%) | 0.829 |
| Language spoken  English  Mostly English  Only Spanish/other language Mostly Spanish  Spanish and English equally | 796 (69.65%)  154 (13.44%)  137 (9.34%)  48 (3.05%)  71 (4.51%) | 451 (71.08%)  88 (14.14%)  60 (7.02%)  26 (2.87%)  40 (4.89%) | 345 (67.88%)  66 (12.57%)  77 (12.24%)  22 (3.27%)  31 (4.04%) | <.001 |
| Foreign-born  Yes  No | 317 (23.22%)  889 (76.78%) | 150 (19.43%)  515 (80.57%) | 167 (27.94%)  374 (72.06%) | <.001 |
| Insurance  Private  Medicaid  Medicare  Dual eligible  Other  None | 614 (55.96%)  111 (7.33%)  158 (12.24%)  62 (4.01%)  199 (16.61%)  59 (3.76%) | 332 (54.66%)  47 (5.82%)  95 (13.84%)  36 (4.18%)  141 (13.84%)  12 (1.26%) | 282 (57.58%)  64 (9.21%)  63 (10.23%)  26 (3.99%)  58 (12.08%)  47 (6.89%) | <.001 |
| Breast cancer risk  More likely to get cancer  Les likely  About as likely | 48 (4.72%)  589 (49.80%)  501 (45.49%) | 29 (5.49%)  332 (51.18%)  273 (43.33%) | 19 (3.72%)  257 (48.02%)  228 (48.25%) | 0.164 |
| CRC risk  More likely to get cancer  Less likely  About as likely | 17 (1.03%)  632 (56.55%)  475 (42.42%) | 8 (0.83%)  365 (58.46%)  253 (40.71%) | 9 (1.29%)  267 (54.10%)  222 (44.62%) | 0.275 |
